# Study protocol for valuing EQ-5D-3L and EORTC-8D health states in a representative population sample in Sri Lanka

**DOI:** 10.1186/1477-7525-11-149

**Published:** 2013-09-02

**Authors:** Sanjeewa Kularatna, Jennifer A Whitty, Newell W Johnson, Paul A Scuffham

**Affiliations:** 1 Centre for Applied Health Economics, School of Medicine, Griffith University, University Drive, Meadowbrook, Queensland, Australia; 2 Population and Social Health Research Programme, Griffith Health Institute, Griffith University, Brisbane, Australia

**Keywords:** Low and middle income countries, Utilities, Health state valuation, EQ-5D, EORTC-8D, Time trade-off, QALY

## Abstract

**Background:**

Economic evaluations to inform decisions about allocation of health resources are scarce in Low and Middle Income Countries, including in Sri Lanka. This is in part due to a lack of country-specific utility weights, which are necessary to derive appropriate Quality Adjusted Life Years. The EQ-5D-3L, a generic multi-attribute instrument (MAUI), is most widely used to measure and value health states in high income countries; nevertheless, the sensitivity of generic MAUIs has been criticised in some conditions such as cancer. This article describes a protocol to produce both a generic EQ-5D-3L and cancer specific EORTC-8D utility index in Sri Lanka.

**Method:**

EQ-5D-3L and EORTC-8D health states will be valued using the Time Trade-Off technique, by a representative population sample (n = 780 invited) identified using stratified multi-stage cluster sampling with probability proportionate to size method. Households will be randomly selected within 30 clusters across four districts; one adult (≥18 years) within each household will be selected using the Kish grid method.

Data will be collected via face-to-face interview, with a Time Trade-Off board employed as a visual aid. Of the 243 EQ-5D-3L and 81,290 EORTC-8D health states, 196 and 84 respectively will be directly valued. In EQ-5D-3L, all health states that combine level 3 on mobility with either level 1 on usual activities or self-care were excluded. Each participant will first complete the EQ-5D-3L, rank and value 14 EQ-5D-3L states (plus the worst health state and “immediate death”), and then rank and value seven EORTC-8D states (plus “immediate death”). Participant demographic and health characteristics will be also collected.

Regression models will be fitted to estimate utility indices for EQ-5D-3L and EORTC-8D health states for Sri Lanka. The dependent variable will be the utility value. Different specifications of independent variables will be derived from the ordinal EQ-5D-3L to test for the best-fitting model.

**Discussion:**

In Sri Lanka, a LMIC health state valuation will have to be carried out using face to face interview instead of online methods. The proposed study will provide the first country-specific health state valuations for Sri Lanka, and one of the first valuations to be completed in a South Asian Country.

## Background

The economic evaluation of health interventions has become an integral part of health policy and decision making for the allocation of limited healthcare resources [1]. The objective of economic evaluation is to provide information on the efficiency of health interventions. An economic evaluation has to identify, measure, value, and compare the costs and consequences of the alternatives being considered. Many high income countries perform economic evaluations and health technology assessments to facilitate informed decisions to allocate healthcare resources. The common outcome measures used in health economic evaluations are Quality Adjusted Life Years (QALYs) and Disability Adjusted Life Years (DALYs). However, most western European and Australian health advisory institutions recommend QALYs as the preferred outcome measure [2-4]. Moreover, unlike DALYs, QALYs use country-specific utility weights. Utility weights are derived from the preferences of a population sample for being in a given health state. Preference elicitation exercises to derive utility weights ideally use health states described by Multi Attribute Utility Instruments (MAUIs). Examples of MAUIs are the three and five level EUROQoL 5 Dimensions (EQ-5D-3L the EQ-5D-5L) [5,6], Short Form 6D (SF-6D) [7], three Health Utilities Indices [8], and Assessment of Quality of Life (AQoL) [9]. Commonly used preference elicitation techniques to value the health states of the MAUIs are the Time Trade-off (TTO), Standard Gamble (SG) and, more recently, Discrete Choice methods [10].

### Low and middle income countries and Sri Lanka

The World Bank classifies countries according to their annual per capita income as low income (US$ 1025 or less in 2011), lower middle income (US$ 1026–4035), upper middle income (US$ 4036–12,476), and high income (above US$ 12,476) [11]. A Low Middle Income Country (LMIC) is defined as a country with low, lower middle or upper middle income [11]. In LMICs such as Sri Lanka, the availability of utility weights is of considerable importance as these countries require greater efficiency of health care resource allocation due to scarce resources and a high disease burden [12]. The availability of utility weights could facilitate cost-utility analyses (CUA) and consequently improved efficiency in allocating health resources in these countries. Utility weights, derived from the preferences of a population of a given country, have been reported to be different from those derived from other countries [13,14]. Moreover, widely different socioeconomic, cultural and social conditions between high income countries and LMICs make it imperative that country-specific utility weights be derived and used in economic evaluations.

A recent literature review showed there have been few health state valuations undertaken in LMICs, impeding the development of CUAs in these countries [15]. Where health state valuations have been undertaken, the EQ-5D-3L is the most commonly applied MAUI to describe health states in valuation studies in the LMIC context. The use of validated methodologies and increased collaboration between high income countries and LMICs is required to build capacity for health state valuation and health economic research in LMICs [15]. Sri Lanka, a South Asian country, does not have any health state valuations for MAUIs available to date. Developing a utility value set for Sri Lanka is important to promote economic evaluations that are based on country-specific preferences. In addition, developing a value set by trialling a feasible, valid methodology for health state valuations in a LMIC environment is an important step forward in progressing knowledge to enable further health state valuations to be undertaken in the LMIC context.

### Barriers to health state valuation in LMICs

LMICs such as Sri Lanka need to improve the efficiency of their health systems. Health state valuation is a crucial element to improve health outcomes to identify efficient healthcare interventions. However, it is challenging for a country like Sri Lanka to undertake health state valuation exercises as it lacks health economics expertise. Further, unlike a high income country, health state valuations cannot be undertaken in LMICs using online surveys due to low internet use and low computer literacy. Thus, health state valuations must involve a community sample and manual data collection processes utilising trained data collectors. This can involve large distances travelled over difficult terrain to reach diverse communities to ensure representativeness of the sample to the population. Nevertheless, low research costs in LMICs facilitate such data collection exercises, provided the necessary expertise is available for research leadership and collaboration. Another perceived obstacle is the potential difficulty for the average person from a LMIC to understand the concept of a trade-off of life in health state valuations. That is, there may be concerns about religious and cultural acceptability of trades between health states and life. Given the lack of available health state valuations and economic expertise in Sri Lanka the readiness of policy makers and the clinical fraternity to accept the concept of utility weights in healthcare decision making is as yet unproven.

### The need for LMIC specific utility value sets

Despite the potential barriers to undertaking valuation studies in LMICs, the development of health state valuations in Sri Lanka is a necessary step to enable an expansion and understanding of the potential role of economic evaluation in health care decision-making. Although the common availability of DALY weights for LMICs may raise the question of why LMICs need utility weights for MAUIs, it is important to remember that the weights developed for use to derive DALYs are not country specific [16]. Yet, resource allocation decisions within a LMIC are country specific. Therefore, country specific utility weights for MAUIs are necessary to derive QALYs to guide resource allocation decisions within any one country, such as Sri Lanka. It is well known that utility weights differ even between high income countries [13] and arguably might be expected to differ to a greater extent between high income countries and LMICs. Preferences to avoid a health state can depend on the health care available, the culture and social support [17]. For these reasons, country specific weights reflecting the preferences of the population are required for LMICs including Sri Lanka.

### Generic vs disease specific multi-attribute utility instruments (MAUIs)

There are situations where generic MAUIs like the EQ-5D-3L cannot be applied. A major issue is lack of sensitivity of generic MAUI for some disease conditions like cancer [18]. As a result, disease specific MAUIs have been validated [18]. Sri Lanka has a high disease burden of cancer [19], for which this protocol describes valuation of health states of cancer specific MAUIs alongside the EQ-5D-3L. Generic instruments are more suitable to calculate utility weights for CUA. Disease specific instruments would help in the rational allocation of resources within a disease, and patients and clinicians specialising in that disease could use these tools to evaluate treatment regimens and any improvements in quality of life. In contrast, generic instruments would help decision makers to make decisions on healthcare resource allocation nationally.

### Aim of the study

To date, no health state valuation study has been undertaken in Sri Lanka or any other South Asian country using a generic MAUI for a representative population sample. Thus, this protocol reports the methods for a health state valuation study to be undertaken in Sri Lanka. The study will derive utility weights for the health states described by the EQ-5D-3L [20], a generic MAUI, and the EORTC-8D [18], a cancer specific MAUI. Moreover, the proposed study will facilitate future research exploring the theoretical question of differences between utilities produced from health states of generic and disease specific MAUIs.

## Methods

The time trade-off (TTO) and standard gamble (SG) [21] are the most widely used methods to value health states [10]. A visual analogue scale (VAS) has also been used to produce national value [22] sets, but this does not involve a trade-off. Dolan *et al*. [23] concluded that the TTO fared slightly better than SG in valuing EQ-5D. Since 1995, the majority of EQ-5D valuations have used the TTO as their preferred elicitation method. Moreover, whilst both TTO and SG are complex tasks, Torrance *et al*. [24] concluded that the TTO is more easily administered to the general public. More recently, the discrete choice experiments (DCE) method has been used to value health states [6,10,25]. However, there is as yet little experience using the DCE to produce a national value set even in high income countries. Consequently, the TTO method will be used to elicit preferences from a representative general population sample. Ethical clearance for this study has been granted by the Griffith Human Research Ethics Committee as well as the Sri Lanka Medical Association Ethics Committee.

### EQ-5D-3L

The EQ-5D-3L has been widely used to describe and value health states in a large number of countries including the UK [23], Spain [14], Germany [26], Australia [20], USA [27], Japan [28], Argentina [29], Chile [30], Thailand [31] and Zimbabwe [32]. Moreover, the reliability and validity of the EQ-5D-3L as a MAUI has been widely established [13,33-38]. The EQ-5D-3L has a total of 243 health states making it relatively easy for valuation; a recent study showed it is feasible to directly value almost the full set of EQ-5D-3L health states [20]. A health state described using the EQ-5D-3L is relatively easy to understand for the participant as it has only five dimensions and three levels in each dimension. Using EQ-5D-3L MAUI full health is described as 11111, stating there are no problems in mobility, personal care, usual activities, no pain/discomfort and no anxiety/depression. The worst imaginable health state according EQ-5D-3L is 33333. In 33333, a person is confined to bed, unable to wash or dress self, unable to perform usual activities, has extreme pain and discomfort and is extremely anxious or depressed.

### EORTC-8D

Parallel use of the EORTC-8D is proposed in this study to inform the debate on the lack of sensitivity of EQ-5D-3L for conditions like cancer. The EORTC-8D is a MAUI for cancer newly derived from the EORTC QLQC-30 questionnaire [18]. It has eight dimensions with four or five levels in each dimension. The EORTC-8D can produce 81,290 health states. Out of this large number, only 84 health states will be valued in the present study [18]. EORTC-8D health states will be divided into seven groups of 12. Each participant will value the allocated seven health states plus immediate death. Only one value set from the UK is currently available for the EORTC-8D [18].

### Selection of health state for valuation

Out of the 243 EQ-5D-3L health states, 196 (plus 11111 and 33333) will be directly valued using TTO methods [20]. A state of “immediate death” will also be used in the valuation. Excluded health states were considered implausible if level 3 on mobility was combined with level 1 on either usual activities or self-care [20]. The selected 196 health states were randomly divided into 14 groups each containing 14 health states, with each participant valuing one group of 14 health state, as well as “the pits” (33333), the state of “immediate death” and full health (11111).

### Interview procedure

Health states will be directly valued in a face-to-face interview. Interviews will be conducted by eight trained data collectors. Interviewers will be university students. They will be trained by a health economic research team from Australia. At the start of the interview, the data collector will describe the purpose of the study and obtain informed consent. The participant will then complete a questionnaire consisting of demographic information and past clinical experience. The language will be English or Sinhalese. Subsequently, an EQ-5D questionnaire will be given to the participant, asking them to rate their current health. Participants will also mark the VAS to indicate their current health. It is a thermometer-like scale from 0–100, where 0 is worst imaginable health state for the participant and 100 the best imaginable.

Participants will then asked to rank 16 health states (14 health states plus 33333 and immediate death). They will then be asked to undertake the TTO valuation of 15 health states (14 health states plus 33333). State of full health (11111) will be used to compare with the each valuing health state [39] in the TTO procedure. After the ranking is recorded, health states will be given to the participant in a random order. The TTO procedure will be conducted as explained by Gudex [40].

For each health state, participants will first be given a choice between immediate death or living in the given health state for ten years followed by immediate death [40], to define which states are considered better/worse than death and consequently which side of a TTO board will be used as a visual aid. For states better than death, a middle value of five years will be offered in full health instead of ten years in the given health state. According to each participant’s response, participants will be offered further trade-offs using the outward titration approach [40]. In this approach, the time spent in full health will be increased or decreased in increments of one year. When there is agreement and refusal between two full years midpoint of the two will be offered. As an example, if a respondent agreed to 3 years of full health instead of 10 years in the given health state and refused 2 years of full health, then 2.5 years of full health will be offered as the trade-off. For states better than death, the TTO value is calculated as x/10, where x is the time the participant agrees to spent in full health instead of 10 years in the given health state.

For states worse than death, an alternative of immediate death will be compared with a combination of living in the given health state and full health followed by death. The total duration will be 10 years. If the time spent in the given health state is x and time spent in full health is y , the TTO value is calculated as ((x/10) -1), ensuring negative values given by states worse than death will have a lower bound of −1.

Following the TTO valuation for EQ-5D health states, the data collector and participant will take a 15 minute break and then value the seven EORTC-8D health states (plus immediate death). The participant will first rank the health states in ascending order, and then provide TTO values for states in random order, using a similar process to that described for the EQ-5D-3L states.

### Sampling

A stratified, cluster sampling technique with random selection within clusters will be used to select a representative sample from Sri Lankan population.

### Sample size

The desired precision of results and the representativeness of the sample were both considered to inform the required sample size. Six months will be the smallest time increment used in the TTO valuation of health states. Using 80% power, a sample size calculation was carried out to determine the sample size needed to identify six month increments (Equation 1) [41].

(1) $$N=\frac{2\;SD^{2}}{\mathrm{MCI}D^{2}}\times {\left(\delta \left(\mathrm{Sig}+\mathrm{power}\right)\right)}^{2}$$

In equation 1, N is the sample size and the minimum clinically important difference (MCID) is the minimum TTO increment. Pickard *et al*. [42] reported the MCID for EQ-5D-3L health states as 0.06-0.07 in a study on cancer. However, for the present calculation, a more conservative MCID of 0.05 was used as six months was minimum increment in the TTO (i.e. 0.5/10). Where 0.5 is a half year and 10 is 10 years of time horizon given for health state valuations. Moreover, use of 0.05 gives higher sample size than 0.06 or 0.07. The weighted mean standard deviation (SD) (0.26) of Thailand [31] and Argentina [29] value sets was used for the equation. The meaning of δ is values for significance (Sig) and power is to be assumed from a normal distribution table. By convention, significance was considered at p < 0.05 and power was 80%, and assumed to follow a normal distribution [41]. To compensate for the inefficiency of data being not normally distributed, the power calculation was adjusted by 1/0.95 [41]. Sample size (N) was calculated as 445 with 80% power of detecting a difference of six months in TTO valuations with a 5% significance based on 0.26 SD.

A second sample size calculation (Equation 2) [43] was carried out to determine the sample size needed for the participants to be considered representative of the Sri Lankan population.

(2) $$N=\frac{z^{2}p\left(1-p\right)}{e^{2}}$$

Sample size (N) was calculated as 385 with 95% confidence level (Z), 50% maximum response distribution rate (p), 5% margin of error (e).

Thus, the higher sample size of 445 can be considered as representative of the general population. To compensate for the loss in efficiency from cluster sampling, the sample size was increased by the “design effect” factor (Equation 3) [44]. Thus, the design effect was calculated for the selected sample size of 445 [45].

(3) $$D=1+\left(b-1\right)\rho \;$$

Design effect (D) is increased when the cluster size (b) and/or rate of homogeneity (ρ) is increased. More precisely, ρ is explained as a measure of variability between clusters as compared to the variation within clusters. As a result, if there is reason to assume the condition of interest would produce higher variability between clusters, ρ would increase and the corresponding sample size would increase. Instead, if the condition of interest would produce less variability between clusters, ρ would be closer to zero. In such cases Bennet *et al*. [45] recommend using a value for ρ of 0.02. This recommendation was followed in the present calculation. This is a reasonable assumption because individuals in each cluster will value the same health states.

There is no clear instruction in the literature for choosing a cluster size. When the cluster size increases sample size increases. However, smaller cluster size means more clusters. More clusters present logistic constraints. It was decided to employ a balance between these two extremes by choosing a cluster size of 30, which gives a sample size of 703 for ρ of 0.02. The number of clusters for that sample size is 23 (Table 1).

**Table 1:** Different sample sizes with variable cluster size and design effect for initial sample size of 445 (using the sample size produced from power calculation)

**Cluster size**	**Ρ**		
	**0.02 (n of cluster)**	**0.09**	**0.5**
20	614 (31)	1205	4672
30	703 (23)	1606	6897
40	792 (20)	2006	9122
50	881 (18)	2407	11347

ρ–rate of homogeneity.

In Sri Lanka, the non-response rate in a previous community survey questioning participants comprehensively on health care use has been estimated to be less than 10% [46]. Thus, a non-response rate of 10% was assumed, and the a final sample size of 773, rounded up to 780, will be invited to participate Thus, 26 clusters of 30 will be used to collect data from the Sri Lankan population.

### Sample selection

A stratified, cluster sampling technique with probability proportionate to size (PPS) was used to select study participants [44]. For pragmatic reasons, four districts were selected from the 25 districts of Sri Lanka. These are Colombo, Kalutara, Kandy and Kurunegala. These districts were selected as they represent a good mix of suburban and rural areas and are logistically feasible for the proposed data collection. A district in Sri Lanka is divided into Medical Officer of Health (MOH) areas, each of which is further divided into Public Health Midwife (PHM) areas. PHM areas will be the primary cluster.

According to population proportions, 26 clusters were divided among the four districts. In each district, sampling interval was calculated by dividing the total district population by the number of clusters [45]. Primary clusters were listed alphabetically in each district. Population and cumulative population of primary clusters were listed. Starting with a randomly generated number (below the sampling interval) and sampling interval, systematic selection of primary clusters will be selected for each district. Using this probability proportionate to size (PPS) method, the probability of selecting any cluster varies with the size of the cluster, giving larger clusters a greater probability of selection and smaller clusters a lower probability [27]. PPS produces a sample which is self-weighted, leading to simplified analysis. As the cluster size is a constant [30] in the present study, each household in the population will have an equal probability of being selected.

In each primary cluster (PHM area), random selection was used to select 30 households by electoral address list. People living in institutions, paid accommodation (boarding houses, Inns) and elderly homes will be excluded. If the selected address is found to be from an excluded category, the data collector will be supplied with a new randomly selected address. If the data collector visits a selected address and gets no answer, they will visit again until they are finished with the cluster. If a selected address is permanently unoccupied during that duration or the occupant does not give consent, it is considered a non- participant and no new selection will be carried out in its place.

Only one eligible adult will be systematically selected to participate per household, according to the Kish grid [47] method (Table 2). Eligible adults are those who are 18 years and older, who can give consent and who routinely sleep at the household even if they are absent at the time of the first visit. Eligible adults will be ordered from youngest to oldest, and the participant selected according to the household number and number of eligible participants. After the selection of the interviewee using the Kish grid [47] method the data collector will complete the interview immediately or arrange an appointment at a convenient time for the selected participant.

**Table 2:** Selection of households using Kish grid

**Household no in a given cluster**	**Number of eligible adults per household**					
	**1**	**2**	**3**	**4**	**5**	**6 or more**
1-5	1	1	3	2	5	1
6-10	1	2	1	3	4	2
11-15	1	1	2	4	1	3
16-20	1	2	3	1	2	4
21-25	1	1	2	1	3	5
26-30	1	2	1	4	3	6

### Data collector training

Eight undergraduates studying a health-related degree will be employed for data collection. All interviewers will be trained in ethics and survey methods as a group in a workshop environment. They will be introduced to the questionnaire capturing demographic information, EQ-5D-3L and EORTC-8D instruments, and the TTO technique. They will undergo closely supervised training in TTO health valuation, including experiential practice-based learning over a two week period. At the end of the training data collectors should be able to explain the objective of the study to a participant, explain the consent conditions and carry out the TTO valuation. A trainer will assess each data collector during a pilot study (n = 40), after which the team of investigators and data collectors will discuss and rectify any challenges in the questionnaires, information sheet, and procedure.

### Validity and reliability

The validity of the instruments will be ensured with the use of the validated Sinhalese version of the EQ-5D-3L in constructing EQ-5D-3L health states. The EORTC-8D health states will be constructed with the aid of its parent instrument the EORTCQLQ-C-30 for which a Sinhalese version has been validated in Sri Lanka [48]. Permissions were obtained from the developers to use the validated EQ-5D and EORTC-8D/EORTCQLQC-30 instruments. The selection of instruments for data collection was used after a thorough literature survey which examined contemporary methods of health state valuations in LMIC. Moreover, content validity of the instruments used for data collection was further strengthened by agreement between the research team for the use of proposed health states valuation instruments and other questionnaires used in the survey.

Reliability of the data will be ensured by rigorous training of data collectors in Sri Lanka to ensure they are familiar with the data collection instruments and competent in delivering interviews using the TTO method. Moreover, the data collection will constantly be monitored by the investigators. Investigators will visit 10% of the randomly selected sample after data collection and ensure randomly selected person was actually selected for data collection. At the end of each day, completed forms will be collected by investigators from the data collectors. Furthermore, randomly selected 40% of data entry will be verified by the investigators.

### Plan for analysis

Demographic characteristics of the sample and a descriptive analysis of participants’ own health status and medical conditions will be examined and compared to national data. For each directly valued EQ-5D-3L or EORTC-8D health state, utility values will be calculated for each participant. Data will be tested for normality and assessed graphically. Mean, standard deviation of TTO values and, if applicable, median and interquartile ranges will be reported. Multi-level modelling will be used to examine the design effect of the sample. Intra-class correlation will be examined to determine the percentage of variance explained by each level of the cluster sampling. Generally more than 10% of the variance needs to be in each level to warrant multi-level modelling.

If not, generalised least square models with random effects will be fitted. Models will be fitted separately for utility and disutility as the dependent variable. Main effect model [49], main effects with N3 [49] and main effects with each pairwise interaction [20] will be tested. Models will be compared for goodness of fit using log likelihood, likelihood ratio test, Akaike Information Criteria (AIC) [50] and Bayesian Information Criteria (BIC) [51]. Sri Lankan EQ-5D-3L value set will be estimated from the selected best fitting model. Using the same methods separate models will be specified for EORTC-8D data to estimate EORTC-8D value set for Sri Lanka.

Sub group analysis will be carried out to identify variables that drives health state valuation [13] in general population in Sri Lanka. The effect of religious practices and beliefs, income, education and disease experience in self or close family will be examined.

## Discussion

This study will be one of the first studies to derive a utility value set for either the EQ-5D-3L or EORTC-8D value sets based on the preferences of a representative population from a LMIC. To our knowledge, it will also be the first health state valuation study undertaken in a South Asian country. As such, this study makes two important contributions. First and foremost, it will produce health state value sets for the generic EQ-5D-3L and disease specific EORTC-8D for use in Sri Lanka. Future health economic evaluations can be carried out using the resulting utility weights specific to Sri Lanka, enabling country specific estimates of the cost-effectiveness of health care interventions to be used by health care decision makers. Secondly, by innovatively applying health state valuation methods in the new context of a LMIC (specifically to assess the preferences of a sample of the Sri Lankan population), the current protocol will provide insights into the feasibility of health state valuation methods in the LMIC context.

The protocol outlines the justification for using utility values in LMICs, selection of EQ-5D-3L and EORTC-8D health states, the TTO exercise, sample size calculation, sample selection and approach to face to face interview of participants. The proposed study will contribute to closing the gap in health state valuation between LMICs such as Sri Lanka and high income countries where these studies are now routinely undertaken. This study will support capacity building for health economic research. Moreover, results of the proposed study will produce values from a South Asian lower middle income country that can be compared with utility weights of high income countries and examined for differences. It is proposed that future research will assess both these differences, as well as confirm whether the difference in utility found for generic and cancer specific instruments in high income countries [52] is also found in for Sri Lanka. More importantly, this protocol paves the way for future health state valuations based on the preferences of representative community samples of the general population in LMICs using local expertise and minimum resources.

## Abbreviations

EQ-5D-3L: EUROQoL 5 dimensions; EORTC-8D: The European organization for research and treatment of cancer 8 dimension; LMIC: Low and middle income countries; TTO: Time trade-off; QALYs: Quality adjusted life years; DALYs: Disability adjusted life years; MAUIs: Multi attribute utility instruments; SF-6D: Short form 6D; AQoL: Assessment of quality of life; SG: Standard gamble; CUA: Cost-utility analyses; VAS: Visual analogue scale; N: Sample size; SD: Standard deviation; MCID: Minimum clinically important difference; D: Design effect; b: Cluster size; ρ: Rate of homogeneity; MOH: Medical officer of health; PHM: Public health midwife; PPS: Probability proportionate to size; AIC: Akaike information criteria; BIC: Bayesian information criteria.

## Competing interests

The authors declare that they have no competing interests.

## Authors’ contributions

SK, JW, NJ and PS developed the methodology together. All authors contributed to draft the manuscript. All authors read and approved the final manuscript.
